# *Yersinia pestis* Caf1 Protein: Effect of Sequence Polymorphism on Intrinsic Disorder Propensity, Serological Cross-Reactivity and Cross-Protectivity of Isoforms

**DOI:** 10.1371/journal.pone.0162308

**Published:** 2016-09-08

**Authors:** Pavel Kh. Kopylov, Mikhail E. Platonov, Vitaly G. Ablamunits, Tat’yana I. Kombarova, Sergey A. Ivanov, Lidiya A. Kadnikova, Aleksey N. Somov, Svetlana V. Dentovskaya, Vladimir N. Uversky, Andrey P. Anisimov

**Affiliations:** 1 State Research Center for Applied Microbiology and Biotechnology, Obolensk, Moscow Region, Russia; 2 Saint Petersburg Medical Pediatric University, Saint Petersburg, Russia; 3 Department of Molecular Medicine and Byrd Alzheimer's Research Institute, Morsani College of Medicine, University of South Florida, Tampa, Florida, United States of America; 4 Laboratory of New Methods in Biology, Institute for Biological Instrumentation, Russian Academy of Sciences, Pushchino, Moscow Region, Russia; George Mason University, UNITED STATES

## Abstract

*Yersinia pestis* Caf1 is a multifunctional protein responsible for antiphagocytic activity and is a key protective antigen. It is generally conserved between globally distributed *Y*. *pestis* strains, but *Y*. *pestis* subsp. *microtus* biovar caucasica strains circulating within populations of common voles in Georgia and Armenia were reported to carry a single substitution of alanine to serine. We investigated polymorphism of the Caf1 sequences among other *Y*. *pestis* subsp. *microtus* strains, which have a limited virulence in guinea pigs and in humans. Sequencing of *caf1* genes from 119 *Y*. *pestis* strains belonging to different biovars within subsp. *microtus* showed that the Caf1 proteins exist in three isoforms, the global type Caf1_NT1_ (Ala48 Phe117), type Caf1_NT2_ (Ser48 Phe117) found in Transcaucasian-highland and Pre-Araks natural plague foci #4–7, and a novel Caf1_NT3_ type (Ala48 Val117) endemic in Dagestan-highland natural plague focus #39. Both minor types are the progenies of the global isoform. In this report, Caf1 polymorphism was analyzed by comparing predicted intrinsic disorder propensities and potential protein-protein interactivities of the three Caf1 isoforms. The analysis revealed that these properties of Caf1 protein are minimally affected by its polymorphism. All protein isoforms could be equally detected by an immunochromatography test for plague at the lowest protein concentration tested (1.0 ng/mL), which is the detection limit. When compared to the classic Caf1_NT1_ isoform, the endemic Caf1_NT2_ or Caf1_NT3_ had lower immunoreactivity in ELISA and lower indices of self- and cross-protection. Despite a visible reduction in cross-protection between all Caf1 isoforms, our data suggest that polymorphism in the *caf1* gene may not allow the carriers of Caf1_NT2_ or Caf1_NT3_ variants escaping from the Caf1_NT1_-mediated immunity to plague in the case of a low-dose flea-borne infection.

## Introduction

The outbreaks, epidemics and pandemics of human plague are caused by *Yersinia pestis* subsp. *pestis* strains that possess universal hypervirulence for a wide range of mammals and are ubiquitously distributed [1–4]. The representatives of a more ancestral subsp. *microtus* are endemic within populations of some voles (*Microtus* spp.) and cause only rare sporadic diseases [1, 5] with no human-to-human transmission [5]. Strains of both subspecies can make a proteinaceous capsule first described by Alexandre Yersin [6]. This antiphagocytic capsule [7] is the main component of plague vaccines [8–11] and is the most important target for laboratory diagnosis of plague [12].

Capsule biogenesis is implemented by a conserved chaperone/usher pathway [13]. Caf1 structural subunit is encoded by a 510-nucleotide *caf1* gene. The precursor protein contains 170 amino acids. The typical cleavage site is located between Ala21 and Ala22 residues [14].

Recently it was shown that a single nucleotide substitution found in the bv. caucasica strains Pestoides F [15] and G8786 [16] resulted in Ala48 → Ser48 substitution, while mutations in *Y*. *pestis* E1979001 (bv. antiqua) and F1991016 (bv. orientalis) resulted only in truncation down to 147 and 130 amino-acid residues giving, most likely, non-functional peptides. More recently, sequencing of *caf1* gene from 41 subsp. *microtus* strains isolated from voles and their fleas in Georgia and Armenia indicated that all of them had the same Ala48 → Ser48 substitution (*caf1* NT2, accession no. EF165977), while the strains isolated from gerbils and susliks of the same region carried the gene with a canonic sequence (*caf1* NT1, accession no. EF165976) [17].

In this study, we provide the first evidence that the allele type NT2 (Ser48 Phe117) is unique to the Transcaucasian-highland and Pre-Araks natural plague foci, while a novel NT3 type (Ala48 Val117) is endemic in Dagestan-highland natural plague focus. This is supported by sequencing data on the *caf1* genes from 119 strains of *Y*. *pestis* belonging to seven out of eight biovars of subsp. *microtus* [4]. Our computational analysis revealed that the Caf1 isoforms found in *Y*. *pestis* endemic strains should not make them significantly different in terms of pathogenicity. To test this hypothesis, we evaluated serologic cross-reactivity and cross-protection of the Caf1 isoforms. All the three isoforms could be equally detected by immunochromatography test for plague. When compared to the classic Caf1_NT1_, endemic Caf1_NT2_ and Caf1_NT3_ had lower immunoreactivity in ELISA and lower indices of self- and cross-immunity. However, although a notable reduction in the cross-protection was observed between all isoforms, the polymorphisms in the *caf1* gene may not provide for Caf1_NT2_ or Caf1_NT3_ *Y*. *pestis* strains the possibility to escape from the Caf1_NT1_-mediated plague immunity in the case of a low-dose flea-borne infection.

## Materials and Methods

### Bacterial strains and culture conditions

*Y*. *pestis* intraspecies classification used in this study corresponds to the International Codex of Bacterial Nomenclature [4, 18, 19]. In this study, we used a total of 119 strains of *Y*. *pestis* representing seven out of eight belonging to subsp. *microtus* biovars, such as caucasica (78), altaica (17), qinghaiensis (2), xilingolensis (3), hissarica (4), talassica (4), and ulegeica (11) [4], as well as belonging to the main subsp. *pestis* a vaccine strain EV line NIIEG (bv. orientalis) and a wild type strain 231 (bv. antique). Characteristics of the strains used for testing of serologic cross-reactivity and cross-protection are shown in Table 1. In addition, avirulent bacteria, Pgm^−^ vaccine strain EV, as well as strains C-376pCD1^−^ and C-824pCD1^−^, depleted of the low-calcium-response virulence plasmid were used for all *Y*. *pestis*-derived Caf1 preparations.

**Table 1:** *Y*. *pestis* strains used in this study for Caf1 isolation, serologic cross-reactivity tests, and virulence experiments.

*Y*. *pestis* strain		Relevant characteristics	Source/reference
**subsp. *pestis* bv. antique**			
231	Wild type strain; universally virulent (LD_50_ for mice ≤ 10 CFU, for guinea pigs ≤ 10 CFU); NT1 allele of *caf1*; Aksai mountain natural plague focus # 33		SCPM-O* [20]
**subsp. *pestis* bv. orientalis**			
EV line NIIEG	The Russian vaccine strain (GenBank: JBOL00000000.1); avirulent); NT1 allele of *caf1*; Madagascar		SCPM-O
**subsp. *microtus* bv. caucasica** (0.PE2)			
C-376	Naturally pPst^−^ strain C-376 virulent for voles and mice (LD_50_ for mice ≤ 5.0 × 10^2^ CFU, for guinea pigs ≥ 10^6^ CFU); NT2 allele of *caf1*; Leninakan mountain natural plague focus # 04		SCPM-O
C-376pCD1^−^	pCD1^−^ derivative of naturally pPst^−^ strain C-376; avirulent		The authors' collection
C-824	Naturally pPst^−^ strain C-824 virulent for voles and mice(LD_50_ for mice ≤ 2.0 × 10^3^ CFU, for guinea pigs ≥ 10^6^ CFU); NT3 allele of *caf1*; Dagestan-highland natural plague focus # 39		SCPM-O
C-824pCD1^−^	pCD1^−^ derivative of naturally pPst^−^ strain C-824; avirulent		The authors' collection

*The State Collection of Pathogenic Microbes and Cell Cultures on the base of the State Research Center for Applied Microbiology and Biotechnology (“SCPM-Obolensk”; http://obolensk.org/center/state-collection.htm).

Bacteria were grown at 28°C for 48 h on brain heart infusion (BHI; HiMedia Laboratories) supplemented with 2% agar at pH 7.2. For Caf1 isolation and purification, bacteria were grown at 37°C in New Brunswick Scientific fermenters with working volumes up to 10 L of liquid aerated media. Growth medium was BHI supplemented with 0.5% yeast extract (Difco). Acidity and oxygen levels were controlled with a specified pO_2_ value >10%. Biomasses were harvested by centrifugation after 48 h.

All handling of samples containing live wild-type *Y*. *pestis* isolates was performed in a select agent authorized BSL3 facility under protocols approved by the State Research Center for Applied Microbiology and Biotechnology Institutional Biosafety Committee.

### Sequencing of *caf1* genes

The nucleotide sequence of each *caf1* gene was determined by direct sequencing of the PCR fragment obtained after amplification of the part of *caf1* operon of the corresponding strain. The primers caf1-F (5'-GAATTTGTTCGTGGATTGGA-3') and caf1-R (5'-TTAAAGGAGGGCATAATAGC-3'), both flanking the *caf1* gene, were located within the *caf1A* and YPMT1.85 (similar to a fragment of integrase) genes, respectively, and were used for both fragment amplification and direct sequencing. Determined sequences of the *caf1* genes were deposited to the GenBank (accession numbers KP641181.1-KP641299.1) and compared to reported sequences of this gene in other *Y*. *pestis* strains.).

### Intrinsic disorder analysis

Amino acid sequences of three Caf1 isoforms were analyzed for the effect of the polymorphism on the intrinsic disorder propensities of related proteins. The intrinsic disorder of these Caf1 isoforms were evaluated by three disorder predictors, PONDR® VSL2 [21], which is one of the more accurate stand-alone disorder predictors [21–23], PONDR® VLXT [24], which is not the most accurate disorder predictor but has a high sensitivity to local sequence peculiarities which are often associated with the disorder-based interaction sites [25, 26], and a metapredictor PONDR® FIT [27], which is more accurate than each of its component predictors, PONDR® VLXT [24], PONDR® VSL2 [21], PONDR® VL3 [28], FoldIndex [29], and IUPred [30].

Since intrinsically disordered proteins or proteins with intrinsically disordered regions are frequently involved in protein-protein interactions and molecular recognitions [25, 31–43] and undergo at least partial disorder-to-order transitions upon binding [25, 33, 43–50], these potential disorder-based binding sites can be identified by various computational means, such as the ANCHOR algorithm [51, 52].

### Isolation and purification of Caf1 isoforms

Avirulent bacteria, Pgm^−^ vaccine strain EV, as well as strains C-376pCD1^−^ and C-824pCD1^−^, depleted of the low-calcium response virulence plasmid were used for all *Y*. *pestis*-derived Caf1 preparations. Cell-free Caf1 was extracted directly from the supernatants of *Y*. *pestis* broth cultures and purified by chromatography. Clarified Caf1 supernatant was slowly mixed with 4 M ammonia sulfate (AS) solution to achieve a 1 M final concentration, and precipitate was collected by centrifugation at 15000 × g for 30 min at 4°C. A HiPrep Phenyl FF (High Sub) 16/10 column (GE Healthcare) was used for the initial chromatography step. Prior to sample loading, the column was equilibrated with 5 column volumes of a 20 mM Tris buffer, supplemented with 1 M AS (pH 8.0). Clarified supernatant was loaded and subjected to the following steps: 4 column volumes 1 M AS + 20 mM Tris, pH 8.0; 100%– 0% AS + 20 mM Tris, pH 8.0 over ten column volumes; hold with 0% AS buffer for 4 column volumes. Ten mL fractions were collected for each elution step. All fractions were analyzed using 12.5% SDS-PAGE. The fractions containing protein Caf1 were combined and concentrated by a second passage through a hydrophobic interaction chromatography (HIC) column. Protein was concentrated by a single step elution by 20 mM Tris, pH 8.0. The fractions were desalted on a XK 26/30 column (GE Healthcare) packed with Toyopearl HW-40F chromatographic media (Tosoh Bioscience) and pre- equilibrated with 20 mM Tris, pH 8.0. Ten mL fractions were collected, and purified Caf1 protein was concentrated using Millipore YM-10 membrane for a subsequent storage at -70°C until used.

### Ethics Statement

All protocols for animal experiments were approved by the State Research Center for Applied Microbiology and Biotechnology Bioethics Committee (Permit No: VP-2015/2) and were performed in compliance with the NIH Animal Welfare Insurance #A5476-01 issued on 02/07/2007, and the European Union guidelines and regulations on handling, care and protection of Laboratory Animals (http://ec.europa.eu/environment/chemicals/lab_animals/home_en.htm).

### Mice

Seven week old female BALB/c mice were purchased from Laboratory Animals Breeding Center (Shemyakin and Ovchinnikov Institute of Bioorganic Chemistry, Russia), housed in polycarbonate cages, and maintained in light-controlled (lights on from 7:00 to 19:00) BSL3 room at the State Research Center for Applied Microbiology and Biotechnology. The temperature and the humidity of the animal room were maintained at 22°C ± 2°C and 50% ± 10%, respectively. Mice were given tap water and mouse mixed fodder PK-120 (Laboratorkorm, Russia) *ad libitum* throughout the study. The number of mouse for experiments used the minimum number of the necessity. The mice were divided into all groups randomly. In this study, we have used humane endpoints for the infected animals. According to the animal protocol the mice should be euthanized in the animal survival studies, when they became either of the following: lethargic, dehydrated, moribund, unable to rise, non-responsive to touch, or lost more than 10% body mass. Humane euthanasia, CO_2_ exposure (anesthesia) using compressed CO_2_ gas followed by cervical dislocation has been used by well-trained individuals. We have monitored the health condition of the animals at least twice a day. There was no unexpected death during the entire set of experiments.

### Animal immunization

Mice were randomly divided into four groups (n = 96 per group) and vaccinated subcutaneously (s.c.) with 10 μg of each Caf1 isoform in 0.1 mL PBS (pH 7.2) adsorbed (1:10, w/w) to the vehicle, aluminum hydroxide gel colloidal suspension (Sigma, USA), or only with the vehicle in PBS as a negative control (placebo). After 30 days, the animals were boosted with an identical dose of the same antigen into the same inoculation site.

### Serologic cross-reactivity

Immunochemical specificity of Caf1 isoforms was assessed with the immunochromatographic rapid diagnostic test for plague that utilizes the anti-*Y*. *pestis* Caf1_NT1_ monoclonal antibody F19 (State Research Center for Applied Microbiology and Biotechnology, Russia).

Blood samples were obtained under anesthesia with CO_2_ gas by retro-orbital route by well-trained individuals. Antibody titers were determined by indirect ELISA a day before and 43 days after the second Caf1 immunization individually in five randomly selected animals from each group of 96 mice immunized with one of the Caf1 isoforms, and the mean titer was calculated. Microtiter plates (Greiner Bio-One, Austria) were coated with 100 ng/well of Caf1 in 0.1 M sodium bicarbonate buffer (pH 9.6) overnight at 4°C. Non-specific binding was blocked with 3% gelatin from cold water fish skin (Sigma) in 0.01 M PBS, pH 7.2. Test sera were added using 2.5-fold serial dilutions in 0.01 M PBS buffer containing 0.05% tween-20 (PBST) and incubated for 2 h at 37°C. After four washes with 0.01 M PBST, 100 μl of sheep anti-mouse IgG conjugated to horseradish peroxidase (GE Healthcare) at a dilution of 1:4000 was added for 1.5 h at 37°C. The plates were washed with PBST and 100 μl of 0.01% o-phenylendiamine-H_2_O_2_ was added to each well. The reaction was stopped by the addition of 100 μl of 1 M H_2_SO_4_ per well, and OD was read at 450 nm using EVOLIS Twin Plus System (BIO-RAD, USA). The titer of antibodies was estimated as the maximum dilution of serum giving an OD reading that exceeded the background by 0.1. Background values were obtained from serum samples collected from the animals injected with the vehicle alone.

### Cross-protection

The ability of an antigen isoform to protect an animal from death after administration of a high dose of a virulent strain producing a different isoform of the same antigen, designated Immunity Index (II) was calculated as the ratio:

$$II=LD_{50imm}/LD_{50veh}$$ (1)

where LD_50imm_ is LD_50_ for animals immunized with an antigen under the study; LD_50veh_ is LD_50_ for vehicle-treated animals.

To estimate LD_50_, 45 days after the booster dose, four groups of mice were infected with virulent strains producing either of the Caf1 isoforms (See Table 1). Each group was subdivided into three subgroups of 32 mice that were challenged with 10-fold dilutions of virulent strains of *Y*. *pestis* (231 (NT1), C-376 (NT2), and C-824 (NT3)), (*a*. 2 × 10^3^ to 2 LD_50_; eight mice for a dose) subcutaneously (in the interior thigh). Animals that succumbed to infection were sacrificed and examined bacteriologically to verify that infection was the cause of death. The remaining animals were observed for three weeks. The animals that survived were humanely euthanized.

### Statistical methods

Data on ELISA were expressed as means ±SEM (standard error of the mean). The LD_50_ and a 95% confidence intervals of the virulent strains for immunized and naïve animals were calculated using the Kärber method [53]. Mortality timeframes were recorded, and the mean life to death time span was calculated for each treatment group. Comparison of the survival curves was performed using Log-rank (Mantel-Cox) test. A *P* value below 0.05 was considered to be significant.

## Results

### Comparison of the Caf1 antigen sequence heterogeneity

**Fig 1:**
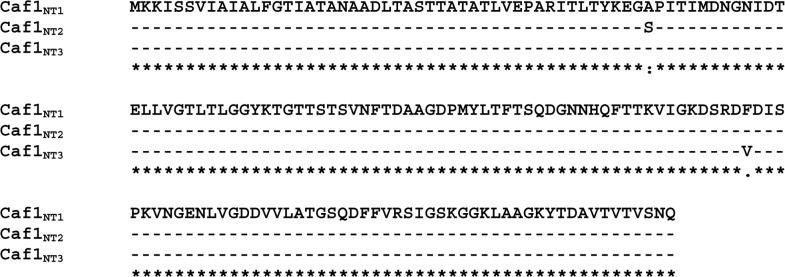
Multiple sequence alignment of the isoforms of Caf1 protein found in different *Y*. *pestis* strains.

Sequencing of *caf1* genes from 119 *Y*. *pestis* strains belonging to different biovars within subsp. *microtus* showed that the Caf1 proteins possess three isoforms, the global allele type NT1 (Ala48 Phe117), NT2 type (Ser48 Phe117) peculiar to Transcaucasian highland and Pre-Araks natural plague foci, and a novel NT3 type (Ala48 Val117) endemic for Dagestan-highland natural plague focus. Fig 1 represents the results of multiple sequence alignment of Caf1 isoforms analyzed in this study and shows that the Caf1_NT2_ found in Transcaucasian highland and Pre-Araks natural plague foci is different from the major allele isoform Caf1_NT1_ by having an Ala48→Ser48 substitution, whereas Caf1_NT3_ protein isolated from the Dagestan-highland natural plague focus has a Phe117→Val117 substitution.

### Intrinsic disorder

**Fig 2:**
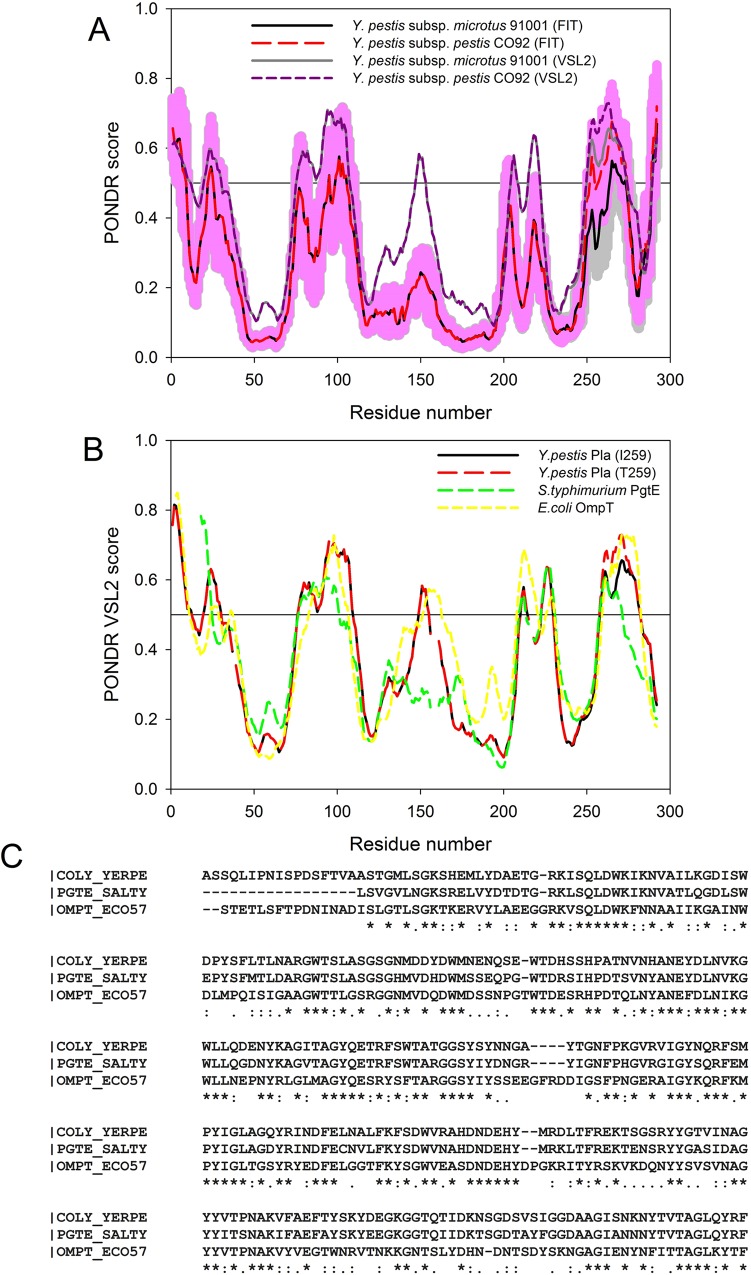
Evaluating intrinsic disorder propensities of different Caf1 isoforms. (A) Disorder profiles obtained for the analyzed proteins by PONDR® VSL2 (Caf1_NT1_ (dashed dark yellow line), Caf1_NT2_ (solid gray line), and Caf1_NT3_ (dotted dark red line)) and PONDR-FIT (Caf1_NT1_ (dashed yellow line), Caf1_NT2_ (solid black line), and Caf1_NT3_ (dotted red line)). Disorder scores above 0.5 correspond to the residues/regions predicted to be intrinsically disordered. Colored shades around the corresponding PONDR-FIT curves represent distributions of errors in evaluation of disorder propensity. (B) Comparison of the disorder profiles obtained for Caf1 isoforms by PONDR VLXT (Caf1_NT1_ (dashed dark yellow line), Caf1_NT2_ (solid gray line), and Caf1_NT3_ (dotted dark red line)) and their intrinsic disorder-based interactability (Caf1_NT1_ (dashed yellow line), Caf1_NT2_ (solid black line), and Caf1_NT3_ (dotted red line)) predicted using the ANCHOR algorithm [51, 52]. To simplify comparison of disorder predisposition and presence of potential disorder-based binding sites, ANCHOR data are present in the (1 –ANCHOR score form). Therefore, in PONDR® VLXT profiles, regions with scores above 0.5 are predicted to be intrinsically disordered, whereas in the ANCHOR profiles, regions with probability below 0.5 are predicted as binding regions.

To understand if found Caf1 polymorphism has an effect on structural and functional properties of this protein, we evaluated the disorder propensities of Caf1_NT1_, Caf1_NT2_, and Caf1_NT3_ isoforms and analyzed the effect of corresponding amino acid substitutions on potential disorder-based binding sites. Results of these analyses are summarized in Fig 2 that compares the disorder profiles obtained for the Caf1 isoforms by PONDR® VSL2 (Fig 2A), PONDR-FIT (Fig 2A) and PONDR® VLXT algorithms (Fig 2B). This analysis revealed that, although Caf1 is predicted to be mostly ordered protein, it has several disordered regions. Curiously, both substitutions found in Caf1 (the Ala48→Ser48 in Caf1_NT2_ and the Phe117→Val117 in Caf1_NT3_) cause noticeable increase in the local intrinsic disorder propensity of the short regions surrounding the corresponding substitutions. Importantly, Fig 2 clearly shows that although the effects of these substitutions on the intrinsic disorder propensities of the Caf1 isoforms are not very strong, there is a reasonable agreement between the results obtained by the three computational tools.

Next, we evaluated the presence of potential disorder-based binding regions in various Caf1 isoforms using the ANCHOR algorithm [51, 52] that utilizes the following criteria: (i) residues of potential disorder-based region belong to a long disordered segment and are not a part of a globular domain; (ii) residues of such a region are not able to form enough favorable contacts with its own local sequential neighbors to fold; (iii) these potential binding residues can form enough favorable interactions with globular proteins upon binding [51, 52]. This algorithm also filters out potential disorder-based regions shorter than six residues. Fig 2B represents the results of this analysis and shows that the Caf1_NT1_ and Caf1_NT2_ have a very short potential binding site (region 140–142) which was filtered out by the algorithm because of its small size. On the other hand, the Phe117→Val117 substitution found in Caf1_NT3_ causes an extension of this site to 4 residues (140–144). Since this length is below the length threshold utilized by ANCHOR algorithm, this potential binding site was also filtered out. Fig 2B also shows that there is some delocalization in the effects of the Phe117→Val117 substitution on the disorder propensity and on the disorder-based binding potential.

### Isolation and purification of Caf1 isoforms

Hydrophobic chromatography allowed for isolation of highly purified Caf1_NT1_ Caf1_NT2_ isoforms as peaks at 350 mM of the ammonium sulphate gradient, while the Caf1_NT3_ elution was spread between 600 and 50 mM under the same elution conditions.

### Serologic cross-reactivity

Serologic cross-reactivity has been tested by both immunochromatography and ELISA techniques. The detection limit of immunochromatography was 1.0 ng/mL with the range of 1.0 ng-1.0 μg/mL. Results obtained were identical for all the three isoforms, indicating that all of them could be readily detected by the antibody used in this method.

**Fig 3:**
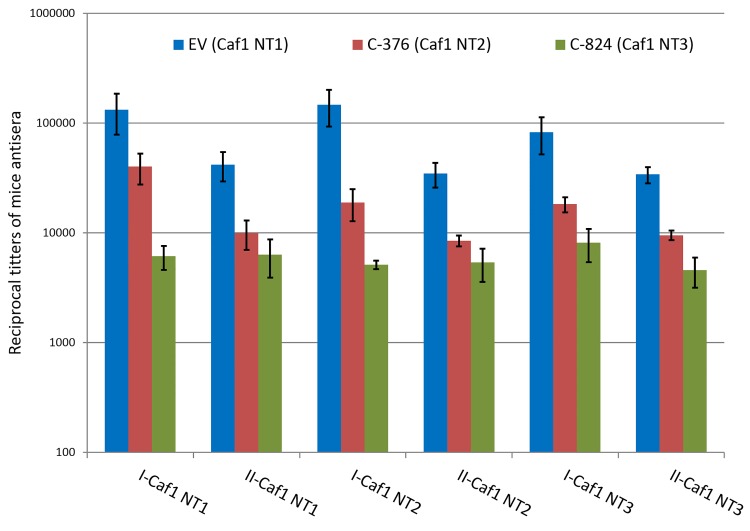
Caf1 isoform cross-reactivity. Mice were immunized with NT1 (blue bars), NT2 (red bars) or NT3 (green bars) and then bled on day 29 after first (I) or day 43 after second immunization (II) and sera samples were tested in ELISA against NT1, NT2 or NT3 isoforms. Data are means ±SEM.

Fig 3 shows that, after both steps of immunization, the antibody titers estimated by ELISA were 4–7 times higher in the animals immunized by the Caf1_NT1_ isoform, regardless of the antigen adsorbed on the plates. The antibody response to immunization with Caf1_NT3_ isoform was the lowest. All the three isoforms absorbed to the plastic were recognized by the sera of immunized animals.

Interestingly, the booster immunizations resulted in a somewhat lower antibody response.

### Cross-protection

**Fig 4:**
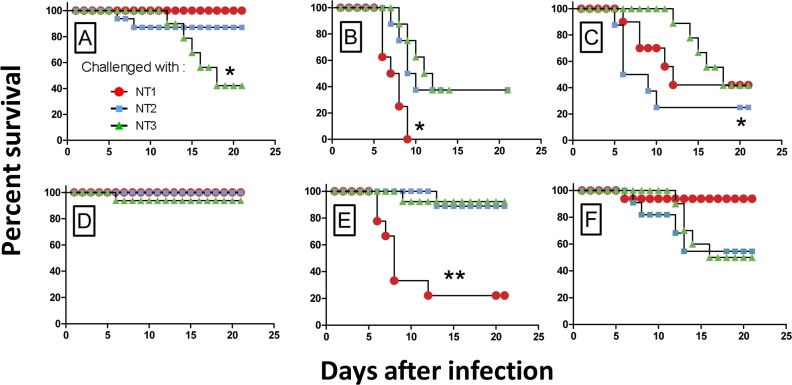
Survival of immunized mice in response to bacterial challenge. Groups of 8 BALB/c mice that were immunized with Caf1_NT1_ (A, D), Caf1_NT2_ (B, E), or Caf1_NT3_ (C, F) isoforms were challenged with *Y*. *pestis* strains producing different Caf1 isoforms: Caf1_NT1_ (circles); Caf1_NT2_ (squares); or Caf1_NT3_ (triangles)), at high (2000 LD_50_, panels A-C), or low (200 LD_50_, panels D-F) doses. Survival was monitored for 21 days after the infection. *P<0.05; **P<0.01 (Log-rank Mantel-Cox test). The results have been acquired with n = 8 BALB/c for each dose of subcutaneous infection.

The results of cross-protection testing are shown in Fig 4 and Table 2. Since the strain 231 was significantly more virulent than the strains C-376 and C-824, the animals were given equal numbers of corresponding LD_50_. The NT1 isoform that has been traditionally used in commercial and experimental vaccines proved to be the most protective one. This isoform was 100% protective in mice challenged with 2000 LD_50_ of the strain 231 (100%), 85% when C-376 strain was used, and 40% mice were protected when infected with the C-824 strain (Fig 4). Thus, immunization with NT1 isoform partly protected against infection induced by NT1 and NT2 carriers even at a high dose. Vaccination with NT2 protected from infection induced by NT2 and NT3, but was less effective against NT1 carriers. At the dose equal to 20 LD_50_ of any of the three strains tested, all animals vaccinated with Caf1_NT1_ survived. This data suggests that vaccination with the NT1 isoform of Caf1 provides a better protection against all the three *Y*. *pestis* variants. All the vehicle-treated mice died within a week after infection regardless of the bacterial stain (data not shown).

**Table 2:** Indices of immunity (II) induced by the three Caf1 isoforms.

Challenge with *Y*. *pestis* strain	Virulence in vehicle-treated mice		Virulence and indices of immunity in mice vaccinated with Caf1 from *Y*. *pestis* strain								
			EV (Caf1_NT1_)			C-376 (Caf1_NT2_)			C-824 (Caf1_NT3_)		
	LD_50_ (c.f.u.)*	Mean time to death	LD_50_ (c.f.u.)	Mean time to death	II	LD_50_ (c.f.u.)	Mean time to death	II	LD_50_ (c.f.u.)	Mean time to death	II
		(days)*		(days)			(days)			(days)	
**231**	**6**	4.6±0.6	**>3.2×10**^**4**^	NA	***>5334***	**562**	9.2±1.0	94	**3.2×10**^**3**^	7.7±1.4	527
(Caf1_NT1_)	1÷22					141÷2.2×10^3^			7.9×10^2^÷1.3×10^4^		
**C-376**	**178**	7.8±1.3	**7.1×10**^**5**^	6	3996	**2.2×10**^**5**^	9.3±1.4	***1264***	**4.0×10**^**4**^	7.8±1.3	225
(Caf1_NT2_)	45÷708		2.2×10^5^÷5.6×10^6^			5.6×10^4^÷1.1×10^6^			1.0×10^4^÷1.6×10^5^		
**C-824**	**10**^**3**^	6.3±1.0	**1.1×10**^**6**^	12.0±2.7	1125	**1.1×10**^**6**^	9.3±1.0	1125	**2.0×10**^**6**^	13	***2000***
(Caf1_NT3_)	251÷3981		2.8×10^5^÷5.6×10^7^			2.8×10^5^÷5.6×10^7^			5.0×10^5^÷1.3×10^7^		

*Values are given as means ±95% confidence intervals.

Vaccination with the Caf1_NT1_ isoform produced a level of protection that exceeded 5334 times that of control (vehicle-treated) mice. The other two isoforms were less potent resulting in only a 1264- to 2000-fold increase in self-resistance. Thus, calculated indices of immunity are in agreement with the data obtained from the survival curves, suggesting that Caf1_NT1_ vaccination is superior compared to the other two isoforms.

## Discussion

Recently, it has been demonstrated that some of *Y*. *pestis* strains isolated from Georgian and Armenian natural plague foci had the NT2 allele of *caf1* gene differing from the typical NT1 allele by a single nucleotide replacement causing a substitution of alanine by serine (Ala48 → Ser48) [15–17]. Our investigations have proven that this replacement is characteristic of all bv. caucasica strains from Transcaucasian-highland plague foci # 4–6 and bv. caucasica isolates from Pre-Araks natural plague focus # 7. It is possible that similar strains are circulating in neighboring territories of Turkey and Iran. We also found a novel NT3 allele (Ala48 Val117) endemic for Dagestan-highland natural plague focus # 39. But the classic Caf1_NT1_ isoform (Ala48 Phe117) remains the major type within the rest of *Y*. *pestis*.

*In silico* analysis of amino-acid sequence of Caf1_NT1_ performed in several laboratories by different methods aimed at location of B- and T-cell epitopes generated conflicting data [54–57]. Information from the laboratory of Dr. D.N. Rao seems to be the most reliable, as it was confirmed by immunoassays and *in vivo* experiments [54].

Our computational analysis revealed that the Ala48→Ser48 and Phe117→Val117 substitutions found in the Caf1_NT2_ and Caf1_NT3_ isoforms, respectively, have some effects on the local intrinsic disorder propensities of these proteins. Both substitutions cause noticeable increase in the intrinsic disorder propensity, but only latter is expected to have some effect on the disorder-based interactivity of this protein. Even in this case, mutation-induced increase in interactivity is minimal, although behavior of the isoform during hydrophobic chromatography is different: the Caf1_NT3_ elution is spread between 600 and 50 mM of the ammonium sulfate gradient, while Caf1_NT1_ and Caf1_NT2_ isoforms are peaking at 350 mM.

It has been shown that Caf1_NT1_ isoform of capsular protein protects bacteria from phagocytosis [58] and exhausts the complement system by selective activation of C`2 and C`4 components thus preventing complement-mediated opsonization of bacteria [59]. On the other hand, it is one of immunodominant antigens responsible for protective immunity against plague [8, 60, 61]. Accordingly, it has been used as the major molecular target for immunodiagnostics, and as a principal component of the vaccines [8, 60–63]. Important questions of whether Caf1_NT1_-mediated protective immunity can be circumvented by the strains that carry NT2 and NT3 alleles of *caf1* gene, and whether current immunoassays can detect Caf1_NT2_- and Caf1_NT3_-producing bacteria have not been addressed before.

In our cross-protection studies, the level of immune response achieved was described in terms of the immunity index. This index represents the difference in challenge dose required to cause death in immunized versus naïve animals (Table 2). Similar data were obtained from the analysis of survival curves (Fig 4). Taking into account that infected fleas can contain up to 5000 *Y*. *pestis* CFU, but the median number of transmitted bacteria is 82 CFU [2], we can predict that the immunity induced by Caf1_NT1_ isoform is sufficient for protection from a low-dose flea-borne infection caused not only by a strain with the same isoform, but also by isolates producing Caf1_NT2_ and Caf1_NT3_ variants. However, this low dose cross-protection may be insufficient in case of infection with high doses of strains producing Caf1_NT2_ or Caf1_NT3_.

**Fig 5:**
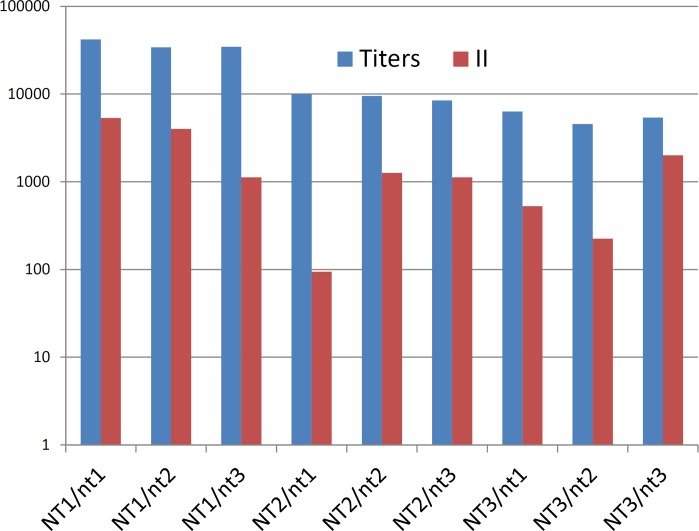
Correlation between serum antibody titers and immunity indices. Capital case–isoforms used for immunization; lower case–genotypes of the strains used for challenge.

The antibody titers positively correlated to the immunity index (Fig 5). For instance, the index of immunity in the Caf1_NT1_ immunized group was higher than that in the other groups, and these mice had higher antibody titers. In contrast, mice immunized with Caf1_NT2_ or Caf1_NT3_ had lower immunity index and lower immunoreactivity in ELISA.

All the *Y*. *pestis* Caf1 protein isoforms tested demonstrated a strong serological cross-reactivity as judged by immunochromatography, suggesting that the monoclonal antibody used in this assay is specific to a shared epitope. It may be reasonable to suggest including the strains with Caf1 endemic isoforms for validation of newly developed immune tests targeting this protein.

The antibody response to the immunizations for all three Caf1 isoforms seems to be lower after the second immunization. We can speculate that this phenomenon may be due to the different terms of antibody-titer measurement after the first and second immunizations, i.e. post-immunization-exposure increase from the four to six weeks was sufficient for onset of depletion of humoral immune response. Another supposition is that the antibody titers measured by ELISA went down after the booster immunizations might be due to the action of regulatory cells. Noteworthy, they were higher after the first vaccination with the NT1 isoform, suggesting that this standard vaccine protein has a superior immunogenicity compared to the minor isoforms. However, after the boost, this difference substantially disappeared, indicating that NT2 and NT3 proteins can also be used for a vaccine, given that immunization is performed in two subsequent injections. These two seemingly less immunogenic isoforms may in fact prove to be better vaccines and induce a longer lasting immunity to *Y*. *pestis* due to a lower capacity to induce regulatory cells [64]. To test these possibilities, more experiments have to be performed.

## Conclusions

The main question addressed in this study was whether specific immunity induced by *Y*. *pestis* Caf1_NT1_ vaccine can be protective against *Y*. *pestis* strains harboring NT2 or NT3 allele types. Our data clearly demonstrate that animals vaccinated with Caf1_NT1_ acquire immunity to all the three bacterial strains tested. Therefore, polymorphism in the *caf1* cannot be considered a sufficient instrument that would allow *Y*. *pestis* an escape from Caf1_NT1_-mediated anti-plague immunity in the case of a low-dose flea-borne infection.
